# Polyphyllin I suppresses human osteosarcoma growth by inactivation of Wnt/β-catenin pathway *in vitro* and *in vivo*

**DOI:** 10.1038/s41598-017-07194-9

**Published:** 2017-08-08

**Authors:** Junli Chang, Yimian Li, Xianyang Wang, Shaopu Hu, Hongshen Wang, Qi Shi, Yongjun Wang, Yanping Yang

**Affiliations:** 1 Longhua Hospital, Shanghai University of Traditional Chinese Medicine, Shanghai, 200032 China; 2 Spine Institute, Shanghai University of Traditional Chinese Medicine, Shanghai, 200032 China; 3 Key laboratory of theory and therapy of muscles and bones, Ministry of Education, Shanghai, 200032 China; 4 School of Rehabilitation Science, Shanghai University of Traditional Chinese Medicine, Shanghai, 201203 China

## Abstract

Osteosarcoma is the most common primary bone cancer in children and adolescents. In spite of aggressive treatment, osteosarcoma has a high mortality rate with minimal improvements in survival over past few decades. Polyphyllin I (PPI), a component in the traditional Chinese medicinal herb Paris *polyphylla Smith*, has been shown to have anti-tumor properties. However, its mechanism as an anti-osteosarcoma agent has not been well elucidated. In this study, we found that PPI suppressed osteosarcoma cell viability, arrested cell cycle in G_2_/M phase, induced apoptosis and inhibited invasion and migration of osteosarcoma cells. Moreover, PPI significantly suppressed intratibial primary tumor growth in xenograft orthotopic mouse model without any obvious side effects. These therapeutic efficacies were associated with inactivation of Wnt/β-catenin pathway, as PPI treatment decreased the amount of p-GSK-3β, leading to down-regulated levels of active β-catenin. PPI induced inhibition of osteosarcoma cell viability was abolished upon addition of GSK-3β specific inhibitor, CHIR99021, while PPI induced inhibition of osteosarcoma cell viability and migration were potentiated by β-catenin silencing. These findings suggested that, *in vitro* and *in vivo*, PPI treatment inhibited osteosarcoma, at least in part, via the inactivation of Wnt/β-catenin pathway. Thus, PPI could serve a novel therapeutic option for osteosarcoma patients.

## Introduction

Osteosarcoma is one of the most aggressive bone cancers in children and adolescents with high metastatic potential^1^. Common sites of osteosarcoma development are near the metaphyseal growth plate located in the long bones of the limbs, including the femur, tibia and humerus. Approximately 40% of the patients present with metastases at the time of diagnosis and, among them, only 20% patients achieve long-term survival^2, 3^. Even though numerous efforts have been made to improve osteosarcoma outcome, including neo-adjuvant chemotherapy and multi-modular therapies, the overall survival rate has failed to improve over the past 20 years^4^. Therefore, there is an urgent need for the development of novel therapeutic strategies for osteosarcoma in order to improve the treatment outcomes.

Polyphyllin I (PPI) is an ethanol extracted from Paris *polyphylla Smith var. yunnanensis* (Franch) Hand-Mazz, also known as Chong-Lou in traditional Chinese medicine, which is a natural herb that has been used in the treatment of infectious disease and cancer in China for thousands years. PPI has been implicated as the active anti-tumor ingredient in Chong-Lou. However, the role and underlying mechanism of PPI-mediated anti-osteosarcoma activity has not yet been fully elucidated.

In the past decade, numerous studies have suggested that the Wnt/β-catenin signaling pathway is one of the major oncogenic pathways involved in osteosarcoma onset and progression^5^. β-catenin, an intracellular signal transducer of the Wnt/β-catenin signaling pathway, has been identified to play a central role in the cadherin protein complex and is crucial for the activation of the Wnt/β-catenin signaling pathway during embryonic development and tumorigenesis^6–8^. In the activated Wnt/β-catenin pathway, Wnt proteins bind to membrane receptors belonging to the Frizzled (Fzd) family, serpentine receptors and low-density lipoprotein receptor-related protein 5/6 (LRP5/LRP6), which are needed to recruit the cytoplasmic phosphoprotein of Disheveled. Disheveled (Dsh/Dvl), the key intermediate in the process, is activated and delivers signals from the formed Wnt/β-catenin receptor complex to the axin and glycogen synthase kinase 3β (GSK-3β) destruction complex to suppress the phosphorylation of β-catenin^9–11^. Wnt protein binding to its receptor results in the accumulation of unphosphorylated β-catenin in the cytoplasm. This accumulated β-catenin then translocates into the nucleus, which subsequently activates its downstream gene targets, such as C-Myc^12^. Additionally, the Wnt signaling pathway regulates various cellular functions, including cell proliferation, apoptosis, invasion and migration, which are all involved in Wnt-dependent carcinogenesis^13, 14^.

The present study aims to further identify the effect and mechanism of PPI on the viability, apoptosis, invasion and migration of human osteosarcoma cells *in vitro* and *in vivo* through its effects on the Wnt/β-catenin signaling pathway.

## Results

### PPI inhibited cell viability of osteosarcoma cells

**Figure 1:**
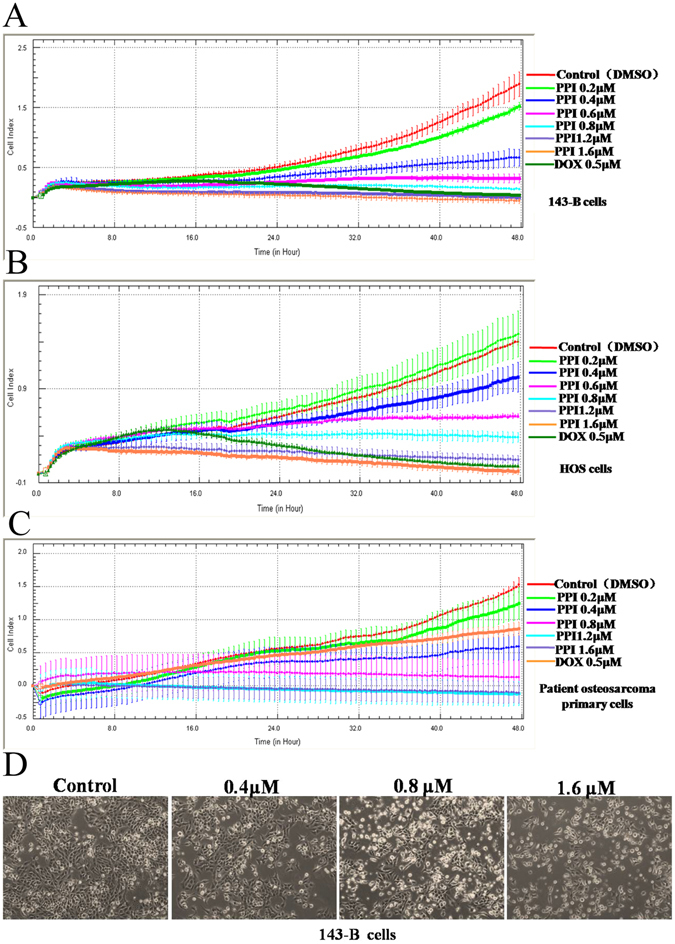
Effects of PPI on viabilities of osteosarcoma cells. Viabilities of osteosarcoma cells were inhibited in dose- and time-dependent manners in 143-B cells (**A**); HOS cells (**B**); patient osteosarcoma primary cells (**C**); The morphological observation of 143-B cells, (**D**) representative images of 143-B cells treated with DMSO (1/10,000V/V) (part 1, control), PPI of 0.4 μM (part 2), 0.8 μM (part 3) or 1.6 μM (part 4) for 24 h, which were observed using an LEICA DMI3000B microscope (100×).

To investigate the effect of PPI on cell viability, the 143-B and HOS cells, and the primary cells from a osteosarcoma patient were challenged with PPI for 48 h, at the final concentration of 0.2, 0.4, 0.6, 0.8, 1.2, and 1.6 μM. DMSO (1/10,000 V/V) only was used in the control group and 0.5 μM doxorubicin (DOX) was used as positive control. The viable cell numbers and IC_50_ of PPI in different cells were analyzed and calculated using xCELLigence RTCA DP system. The results showed that PPI treatment had a strong inhibitory effect on the viability of 143-B (Fig. 1A) and HOS (Fig. 1B) cells, and the patient osteosarcoma primary cells (Fig. 1C), with an IC_50_ values of 0.3942 µM, 0.8145 µM, and 0.5316 µM for 143-B, HOS and patient osteosarcoma primary cells, at the time point of 48 h, respectively. Morphologically, PPI treated 143-B cells gradually became rounded and began to detach from the culture plates in a dose-dependent manner, in comparison with the DMSO control (Fig. 1D). These data indicated the anticancer activity of PPI in osteosarcoma cells.

### PPI induced apoptosis in osteosarcoma cells

**Figure 2:**
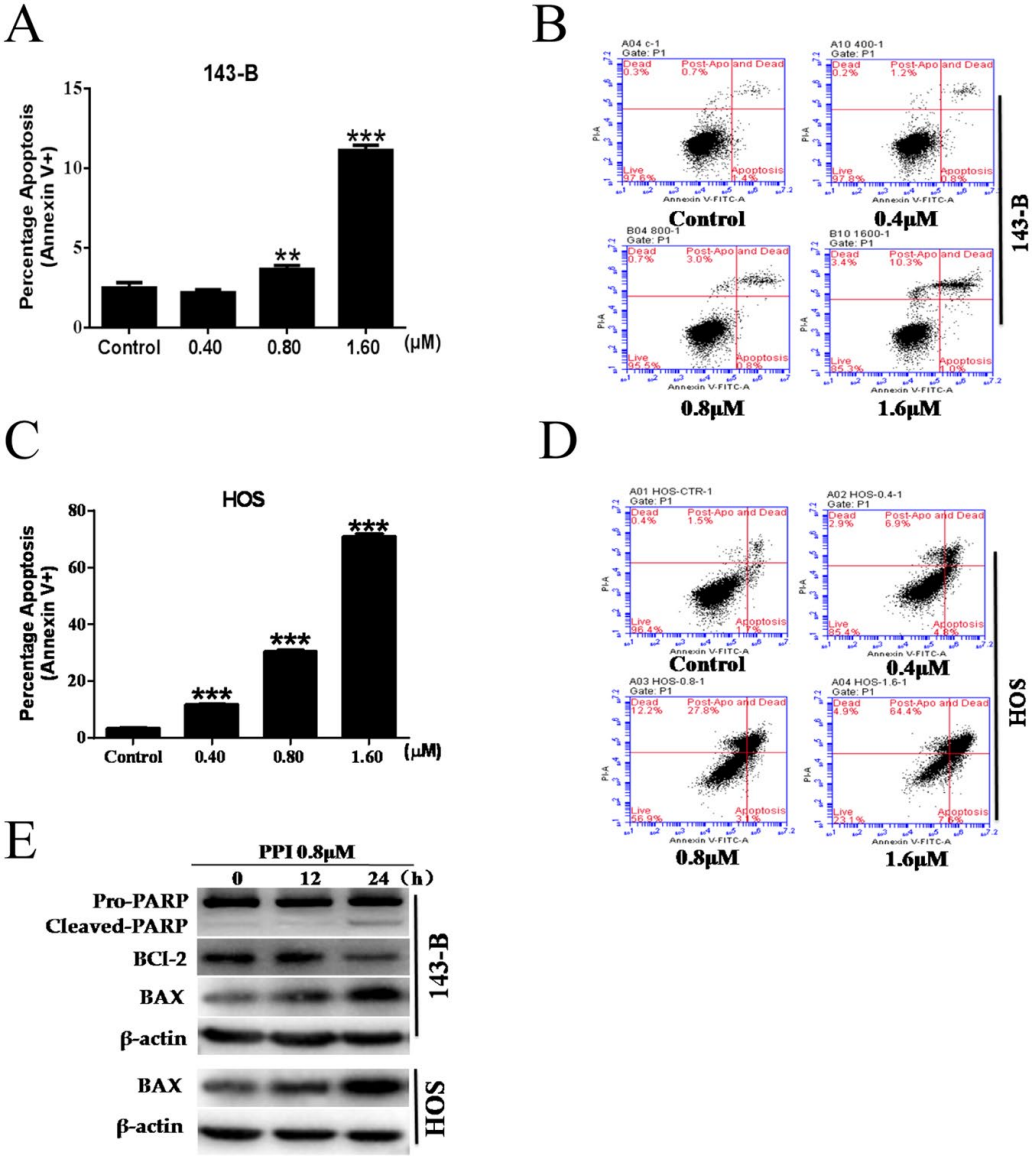
Effects of PPI on osteosarcoma cell apoptosis. (**A**) Percentage of apoptotic 143-B cells; (**B**) representative graphs of apoptotic 143-B cells; (**C**) percentage of apoptotic HOS cells; (**D**) representative graphs of apoptotic HOS cells; (**E**) 143-B cells and HOS cells were respectively treated with 0.8 μM PPI for indicated times, and expressions of test proteins were examined by western blotting analysis, β-actin was used as loading control, and the full-length blots were included in the supplementary information file as Figure S1. *p < 0.05, **p < 0.01, ***p < 0.001, versus control.

In order to determine whether PPI mediated anticancer activity in osteosarcoma cells was associated with the induction of apoptosis, we challenged the 143-B and HOS cells with PPI for 24 h at concentrations of 0.4, 0.8 or 1.6 μM. DMSO (1/10,000 V/V) only was used in the control group. Cells were then quantified by flow cytometry using Annexin V-FITC/PI double staining. As shown in Fig. 2A–D, we found that treatment with PPI resulted in a dose-dependent induction of apoptosis in both 143-B and HOS cells. This was further confirmed by PPI (0.8 μM) induction of a time-dependent increase of cleaved PARP (p89) and BAX, and a decrease in the anti-apoptotic protein of BCL-2 in 143-B cells, and a time-dependent increase of BAX in HOS cells (Fig. 2E).

### PPI induced cell cycle arrest of osteosarcoma cells

**Figure 3:**
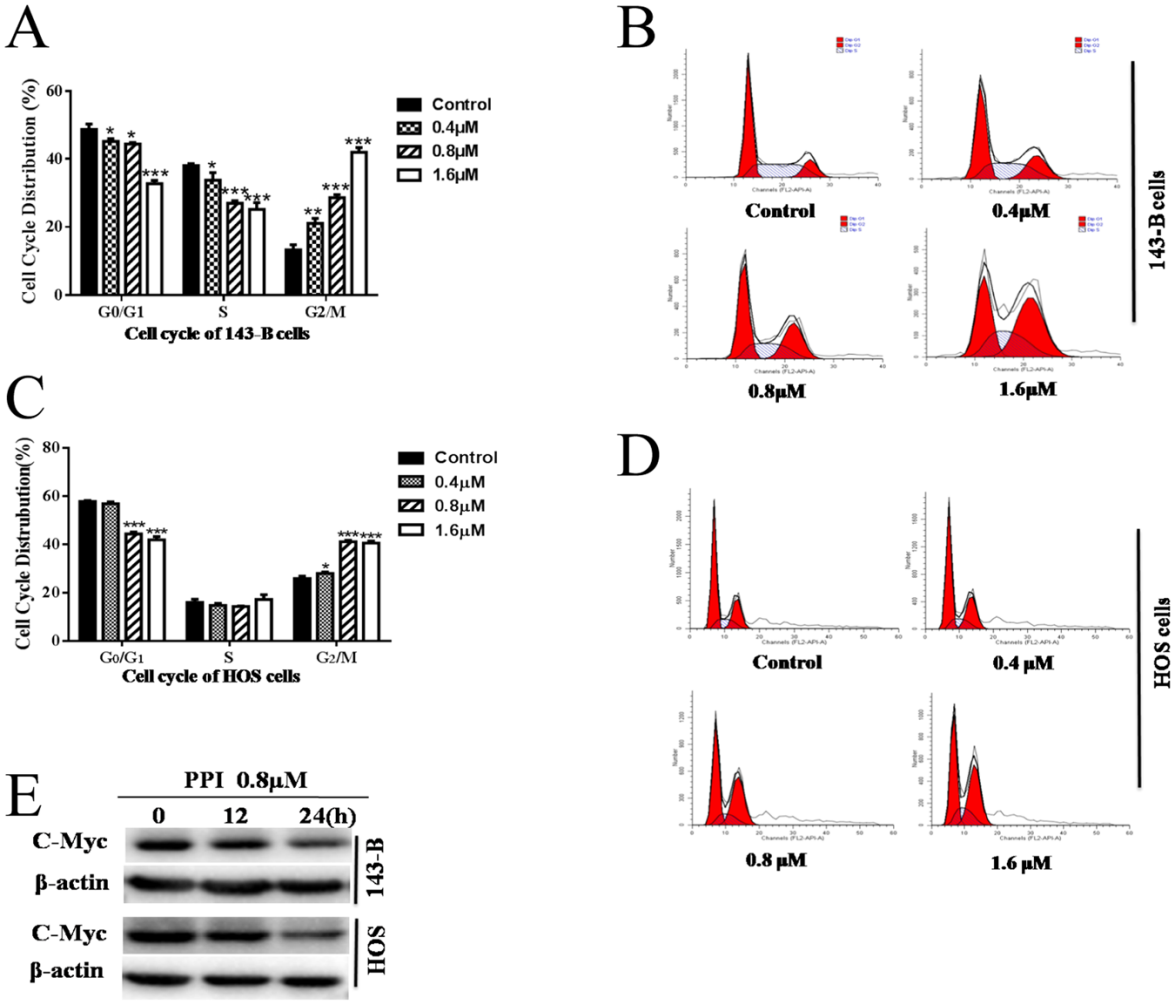
Effects of PPI on cell cycle progression of osteosarcoma cells. (**A**) Percent distribution of specific phases across the 143-B cell cycle; (**B**) representative graphs of cell cycle for143-B cells; (**C**) percent distribution of specific phases across the HOS cell cycle; (**D**) representative graphs of cell cycle for HOS cells; (**E**) 143-B cells and HOS cells were respectively treated with 0.8 μM PPI for indicated times, expressions of C-Myc was examined by western blotting analysis, and β-actin was used as a loading control, and the full-length blots were included in the supplementary information file as Figure S2. *p < 0.05, **p < 0.01, ***p < 0.001, versus the control.

To further investigate the effect of PPI on the osteosarcoma cell cycle progression, 143-B and HOS cells were treated for 24 h with 0, 0.4, 0.8 or 1.6 μM of PPI, and DMSO (1/10,000 V/V) only was used in the control group. The cell cycle distribution was analyzed using the Cell Cycle Detection kit by flow cytometry. PPI-treatment of 143-B and HOS cells resulted in G_2_/M phase arrest, and a corresponding reduction in the amount of cells in G_0_/G_1_ and S phase, in a dose-dependent manner (Fig. 3A–D). This was further evidenced by PPI (0.8 μM) induced down-regulation of C-Myc in 143-B and HOS cells in a time-dependent manner (Fig. 3E). These data suggested that the PPI-mediated inhibition of osteosarcoma cell survival is due to cell cycle arrest and DNA synthesis blockage.

### PPI suppressed the migration and invasion of osteosarcoma cells

**Figure 4:**
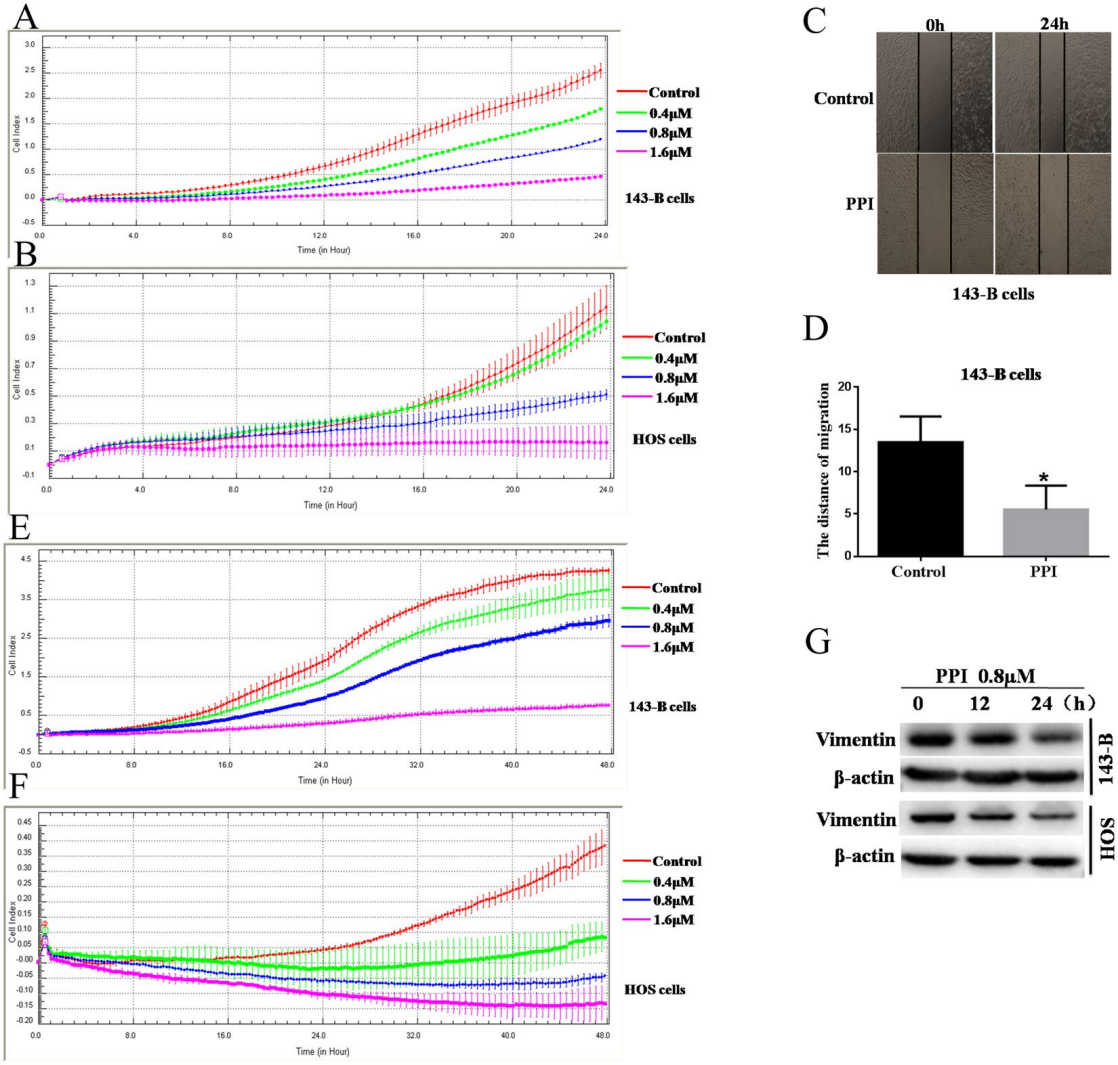
PPI inhibited migration and invasion of osteosarcoma cells *in vitro*. Migration and invasion of 143-B and HOS cells were assessed by xCELLigence RTCA DP system respectively in the presence of 0.4, 0.8, 1.6 μM PPI for 24 h in migration assay and 48 h in invasion assay, and compared with the control group in the presence of DMSO (1/10,000V/V). Results shown were continuous monitor of cells migrated or invaded from the upper chamber through the membrane into the bottom chamber over time, (**A**) migration of 143-B cells; (**B**) migration of HOS cells; (**E**) invasion of 143-B cells; (**F**) invasion of HOS cells. (**C**) representative images of wounds were observed using an LEICA DMI3000B microscope (100×); (**D**) quantitative results of distance migrated. (**G**) 143-B cells and HOS cells were respectively treated with 0.8 μM PPI for indicated times, and the expressions of Vimentin were examined by western blotting analysis, and β-actin was used as a loading control, and the full-length blots were included in the supplementary information file as Figure S3. *p < 0.05, **p < 0.01, ***p < 0.001, versus the control.

The migratory ability of 143-B and HOS cells was determined using an xCELLigence RTCA DP system and wound-healing migration assay. Our results indicated that migration of 143-B (Fig. 4A) and HOS (Fig. 4B) cells was inhibited by PPI in a dose- and time-dependent manner.

This was further confirmed with wound-healing migration assay, as shown in Fig. 4C and D, PPI significantly inhibited the wound closure of 143-B cells (*p* < 0.01).

To investigate if PPI also could inhibit the invasiveness of human osteosarcoma cells, we examined the invasive ability of 143-B and HOS cells after being treated with PPI, through a matrigel-coated filter by xCELLigence RTCA DP system. We found that PPI decreased the ability of 143-B (Fig. 4E) and HOS (Fig. 4F) cells to traverse the matrigel versus the control cells (DMSO only treated), in a dose- and time-dependent manner.

Epithelial-mesenchymal transition (EMT) is a critical mechanism for the development of cancer metastasis, and closely related with migration and invasion of cancer cells. Therefore, we next analyzed specific molecular changes in Vimentin levels associated with EMT by Western blot, using total cell lysates from 143-B and HOS cells, respectively, exposed to PPI (0.8 μM). During EMT, Vimentin expression is activated. PPI-treated 143-B and HOS cells both exhibited a significant time-dependent down-regulation of Vimentin (Fig. 4G). These data supported that PPI treatment could decrease the motility of osteosarcoma cells by reversing the EMT *in vitro*.

### Up-regulation of β-catenin in human osteosarcoma specimens and PPI induced inactivation of Wnt/β-catenin signaling pathway

**Figure 5:**
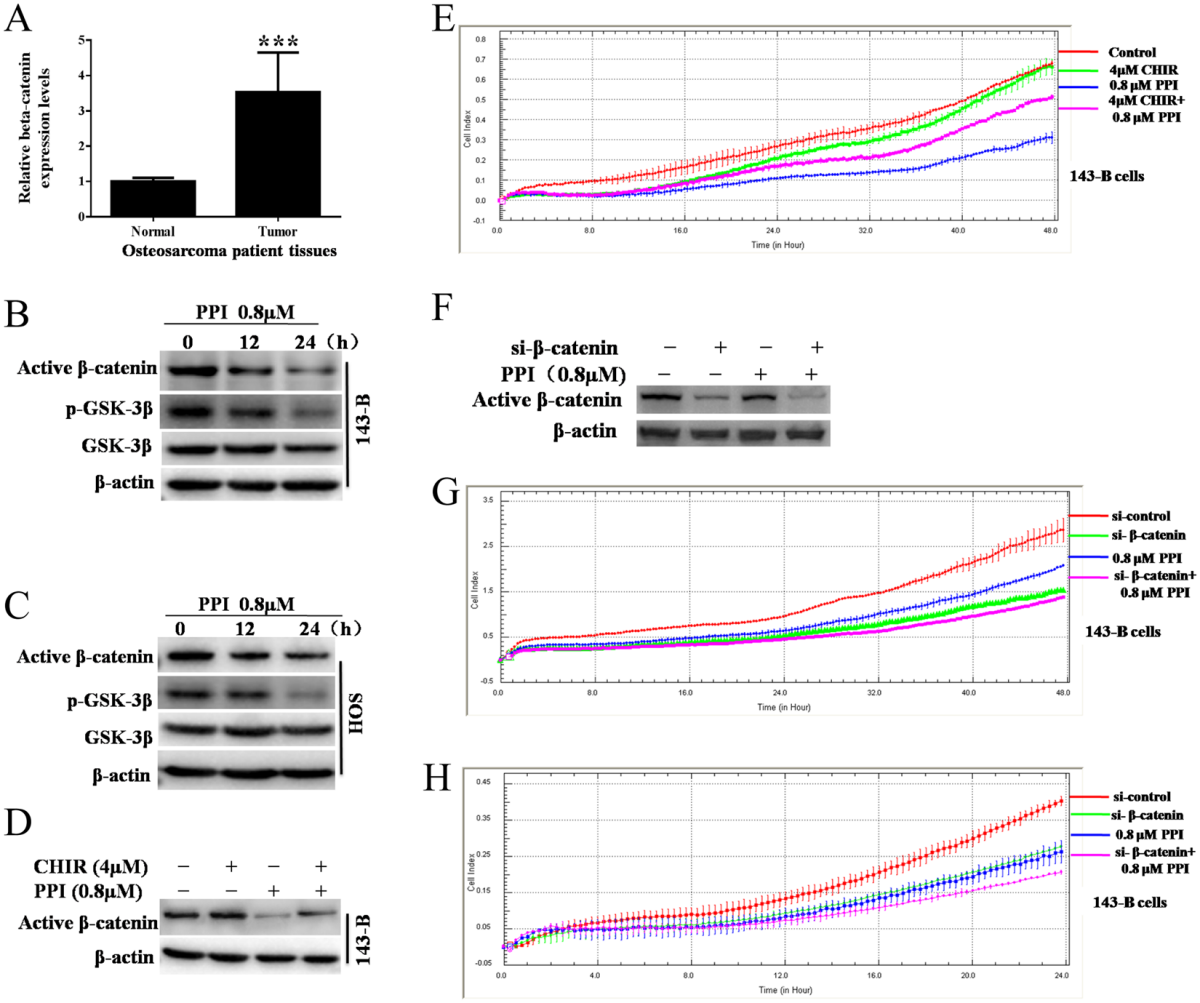
PPI suppressed osteosarcoma cells by specifically inactivating Wnt/β-catenin signaling pathway. (**A**) RT-PCR analysis of β-catenin expression level in matched human osteosarcoma tissues (tumors) and adjacent noncancerous tissues (normal) from 3 patients. (**B**) 143-B cells and (**C**) HOS cells were respectively treated with 0.8 μM PPI for indicated times, and expressions of test proteins were examined by western blotting analysis, β-actin was used as loading control, and the full-length blots were included in the supplementary information file as Figures S4 and S5. 143-B cells were pretreated with 4 μM CHIR9902 (the specific GSK-3β inhibitor) for 24 h before exposed to 0.8 μM PPI for another 48 h, the combined CHIR and PPI treatments result in rescued (**D**) active β-catenin expression (the full-length blots were included in the supplementary information file as Figure S6) and cell viability (**E**) compared to the PPI treatment alone in 143-B osteosarcoma cells. 143-B cells were transfected with either small interfering RNA-targeting β-catenin (si-β-catenin) or si-control for 48 h before exposed to 0.8 μM PPI for another 24 h, si-β-catenin potentiated PPI induced (**F**) down-regulation of active β-catenin expression (the full-length blots were included in the supplementary information file as Figure S7), (**G**) decrease of cell viability and (**H**) inhibition of migration of 143-B osteosarcoma cells induced by PPI. *p < 0.05, **p < 0.01, ***p < 0.001, versus the control.

Wnt/β-catenin signaling pathway has been shown to be associated with cell proliferation, apoptosis, cell cycle regulation, and EMT in osteosarcoma. To demonstrate the up-regulation of β-catenin in human osteosarcoma specimens, we compared the mRNA expression level of β-catenin in matched human osteosarcoma tissues and the adjacent noncancerous tissues from 3 patients, as shown in Fig. 5A. We detected a statistically significant up-regulation of β-catenin mRNA expression levels in human osteosarcoma specimens versus the normal control tissues (p < 0.001). The upstream of β-catenin, glycogen synthase kinase 3β (GSK-3β) destruction complex, suppresses the phosphorylation of β-catenin. Therefore, we further evaluated the effect of PPI on the activation of GSK-3β and β-catenin pathway in osteosarcoma cells. Western blot analysis revealed that PPI inhibited the phosphorylated GSK-3β (p-GSK-3β) and active (non-phospho) β-catenin in a time-dependent manner in both 143-B (Fig. 5B) and HOS (Fig. 5C) cells.

In order to confirm that these anti-osteosarcoma results are specifically achieved by PPI-induced inactivation of Wnt**/**β-catenin, and not due to nonspecific effects of PPI treatment, we pretreated 143-B cells with the specific GSK-3β inhibitor, CHIR99021, before challenging 143-B cells with 0.8 μM PPI. Our results showed that the observed effects of PPI treatment on 143-B cells were abolished by addition of CHIR99021, evidenced by the rescued expression of active β-catenin (Fig. 5D) and cell viability (Fig. 5E). Meanwhile, active β-catenin expression, cell viability and migration were also determined after silencing β-catenin using siRNA-β-catenin in 143-B cells followed by PPI treatments. The results showed that, in 143-B osteosarcoma cells, PPI treatment induced an increased inhibition of active β-catenin protein expression (Fig. 5F), cell viability (Fig. 5G) and cell migraion (Fig. 5H) after β-catenin silencing. These data suggested that PPI inhibits osteosarcoma survival via, at least in part, the inactivation of β-catenin pathway.

### PPI retarded tumor growth in xenograft-bearing nude mice

**Figure 6:**
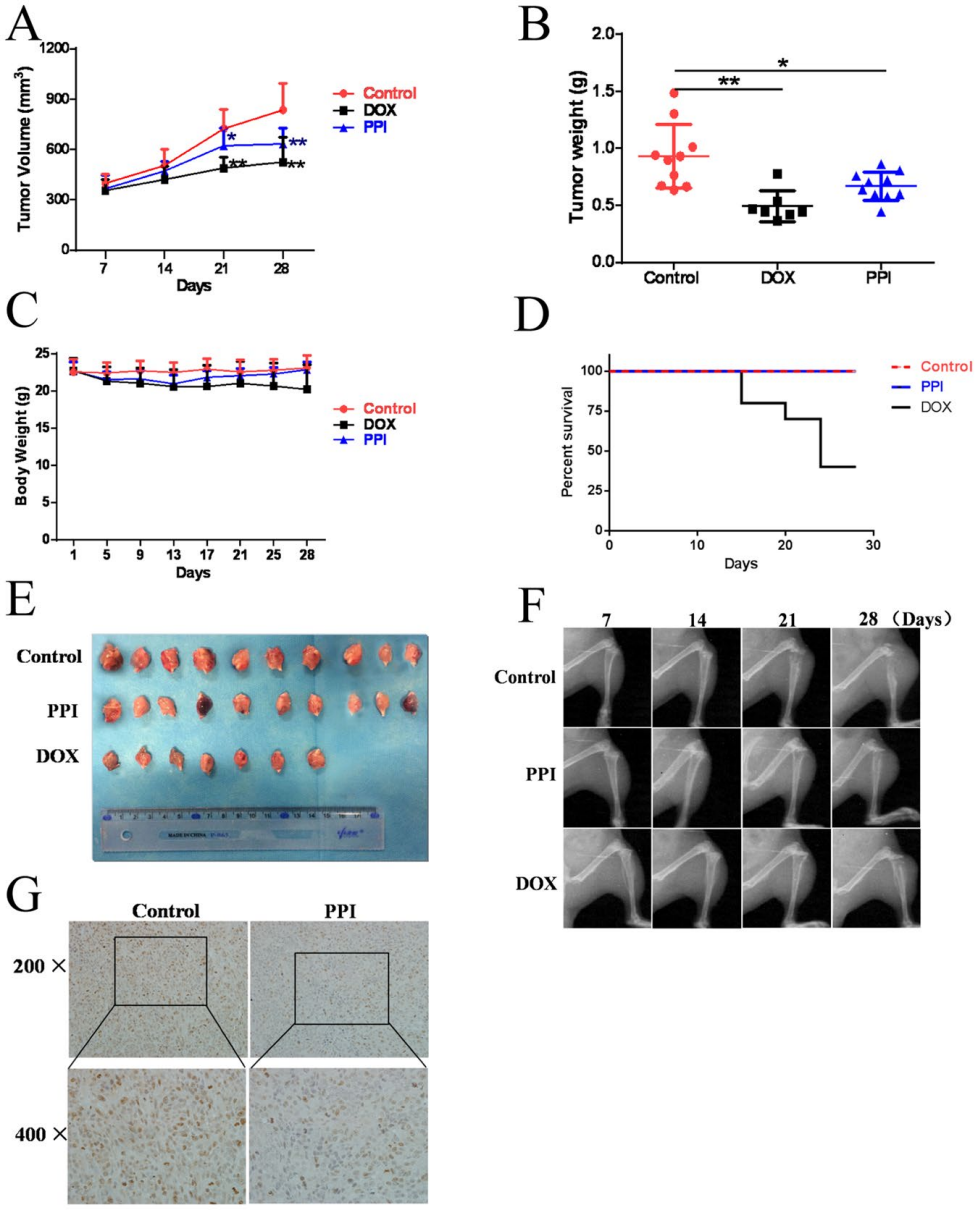
PPI retarded tumor growth *in vivo*. (**A**) Change in tumor volumes (**B**) change in tumor weights; (**C**) change in body weights; (**D**) survival rate of mice, in PPI-treated, DOX-treated and vehicle groups, after the treatment for 28 days. (**E**) Image of all of the xenograft tumors isolated from PPI-treated, DOX-treated and control groups after the treatment for 28 days. (**F**) Tumor growth in the tibia was monitored every week by X-ray (Image Station *In-Vivo* FX system, Kodak, Japan) post 143-B cell transplantation into the tibia. (**G**) Ki-67 immunohistochemistry staining of paraffin embedded 5 micron thick tumor sections of 143-B xenograft-bearing nude mice, images were obtained using an Olympus BX50 microscope. *p < 0.05, **p < 0.01, versus the control.

To further explore whether PPI can suppress osteosarcoma growth *in vivo*, 143-B cells were inoculated directly into nude mice tibias. One day after the inoculation, mice began treatment with either PPI (4 mg/kg·body weight) for the PPI group, same volume of saline with DMSO (1/10,000 V/V) for the control group, or DOX (1 mg/kg) for the positive control group. These treatments were administered by intraperitoneal injection for 28 consecutive days. After 28 days, mice were euthanized to compare tumor burden between the control (saline with DMSO) and PPI-treated group. The results indicated that the PPI treatment significantly retarded tumor growth in nude mice as measured by tumor volume (Fig. 6A) and tumor weight (Fig. 6B), while body weight was unaffected (Fig. 6C), and there was no mice died in PPI group during the period of our experiment (Fig. 6D). Overall, the tumor size in PPI-treated group was markedly smaller compared to the control group (Fig. 6E). This observed ability of PPI to suppress tumor growth was further supported by X-ray of the osteosarcoma lesion in the tibia. As shown in Fig. 6F, Xenograft-bearing legs from untreated mice showed highly invasive tumors that breached the bone marrow barrier and resulted in both bone loss and malignant osteogenesis. In contrast, xenograft-bearing legs from PPI treated mice had modest architecture destruction, the tumors remained confined within the primary bone marrow, and the general bone infrastructure was intact. Ki-67 monoclonal antibody detects only the nuclear antigen in proliferating cells. Ki-67 proliferation index is associated with increased risk of developing metastasis. To further define the inhibitory effect of PPI treatment on tumor growth *in vivo*, Ki-67 expression in xenograft tumor tissues was detected. As can be seen in Fig. 6G, Ki-67 immunohistochemistry staining showed that PPI treatment significantly inhibited Ki-67 expression in tumors, as indicated by the decreased brown nuclear staining. This further confirmed that PPI could also inhibit osteosarcoma cell proliferation *in vivo*.

## Discussion

Although recent advances in different therapeutic approaches have been used to treat osteosarcoma, such as neoadjuvant chemotherapy, surgery and combinational chemotherapy, it is still one of the solid tumors with the highest mortality rate. Most therapeutics are ineffective against osteosarcoma and have severe side effects. Additionally, osteosarcoma frequently acquires multidrug resistance^15, 16^. As previous studies showed, between 25% and 40% of osteosarcoma patients will suffer from a metastatic recurrence. Of these, almost 75% of them will die of the disease within 5 years^17–19^. Thus, there is an urgent need for identification of less toxic and more effective treatments to osteosarcoma.

In this study, we evaluated the effects of PPI treatment on osteosarcoma, both *in vitro* and *in vivo*. Our data demonstrated that PPI effectively inhibited osteosarcoma cell viability and induced apoptosis of osteosarcoma cells in a dose- and time-dependent manner. The results were further confirmed by the up-regulation of BAX and cleaved-PARP, and down-regulation of BCL-2 protein expressions, following exposure to PPI. We further explored the effect of PPI treatment on osteosarcoma cells by examining cell cycle progression. Osteosarcoma cells were significantly arrested at G_2_/M phase via down-regulation of C-Myc protein. As osteosarcoma is highly metastatic, we examined the effect of PPI treatment on processes involved in metastasis, such as cell migration. We found that exposure to PPI inhibited osteosarcoma cell migration and invasion, *in vitro*. EMT is an important mechanism in the regulation of cell invasion and migration. During this process, the disassembly of adherens junctions via EMT is needed to promote the metastatic potential of cancer cells^20^. Thus, we investigated the effect of PPI on EMT in osteosarcoma cells. The results showed that PPI could reverse the EMT process by down-regulating Vimentin expression.

To develop the novel treatment strategies, tumor xenografts have been the most widely used models in a preclinical experiment. However, animal models of osteosarcoma are difficult to establish, due to the mechanical and technical challenges of orthotopically xenografting osseous tissue. Though the existing model systems produced by subcutaneous injections of osteosarcoma are fast, cheap, and reproducible, this model does not sufficiently reflect the clinical condition of human with osteosarcoma^21–23^. Therefore, we used a validated orthotopic implantation mouse model leading to a reproducible and genetically representative xenograft model. The orthotopic xenograft model that we have established can better recapitulate the biology of osteosarcoma, thereby facilitating the research of novel treatment in a model that is more similar to the human primary osteosarcoma^24–26^. Compared to traditional approaches, our orthotopic mouse model is an advantage of this study^27^. Using this model, we found that PPI treatment could inhibit tumor growth in xenograft-bearing nude mice. PPI-treated mice had smaller, less proliferative tumors, as indicated by a significant decrease in tumor size, amount of osteolytic lesions and malignant osteogenesis, and level of Ki-67 expression in tumors.

Our work further confirmed that β-catenin plays a key role in activating the Wnt signaling pathway in osteosarcoma. To further elucidate the mechanism of PPI in inhibition of osteosarcoma growth and progression, we analyzed the amount of active β-catenin in PPI-treated osteosarcoma cells. Active (non-phospho) β-Catenin (Ser33/37/Thr41) (D13A1) Rabbit mAb is designed to specifically recognize the endogenous β-catenin protein when residues Ser33, Ser37 and Thr41 are not phosphorylated by its upstream regulator, GSK-3β, and thus is functionally active in cell-cell adhesion and/or the canonical Wnt signaling pathway. It does not detect β-catenin protein with tri-phosphorylated Ser33/Ser37/Thr41. When GSK-3β is phosphorylated at serine 9, β-catenin cannot be phosphorylated and rendered inactive. We found that treatment with PPI resulted in decreased p-GSK-3β levels, which in turn inhibited the accumulation of active β-catenin. Once phosphorylated, β-catenin can be ubiquitylated and targeted for degradation by the proteasome^28^. Consistent with previous reports, our data demonstrated PPI could inhibit the growth of 143-B cells by inhibiting phosphorylation of GSK-3β, thereby promoting β-catenin phosphorylation by GSK-3β, leading it to be targeted for degradation. In order to show that this is a result of the specificity, we used both the specific inhibitor CHIR99021 of GSK-3β and small interfering RNA-targeting β-catenin (si-β-catenin). However, PPI may potentially interact with multiple signaling pathways in osteosarcoma. Therefore, further investigation is needed to fully elucidate its action mechanisms *in vitro* and *in vivo*.

It has previously been reported that, at the dose of 50 mg/kg, PPI showed a low oral bioavailability (around 0.62%). When co-administrated with verapamil (VPL) or cyclosporine A (CYA), bioavailability increased from 0.62% to 3.52% or 3.79% respectively^29^. Oxidation, glucuronidation and deglycosylation were found to be the main metabolic processes of PPI in rats after intragastric administration. The pharmacokinetics of 3 deglycosylation metabolites of PPI with confirmed anticancer effects, including prosapogenin A, trillin and diosgenin, suggested that, at the dose of 500 mg/kg, the metabolites underwent a prolonged absorption and slower elimination of PPI^30^. These reports provided valuable information on the metabolic fate of PPI, which is helpful in further understanding the mechanism of action. However, the pharmacokinetics of PPI administrated by subcutaneous injection in rats and human have not been reported.

Taken together, our data demonstrated that PPI inhibited osteosarcoma growth *in vitro* and *in vivo* by inducing cell apoptosis, cell cycle arrest and inhibition of invasion and migration, indicating that PPI could potentially be used for the treatment of osteosarcoma. Furthermore, these findings indicated that PPI targeting of the Wnt/ β-catenin signaling pathway could be, at least in part, the mechanism by which PPI inhibits osteosarcoma growth and progression. Additional pharmacological and toxicological experiments are needed to verify this conclusion. In all, our findings indicate that PPI warrants further clinical study in order to clarify its mechanism of action and potential uses in osteosarcoma treatment.

## Materials and Methods

### Compounds and reagents

PPI was purchased from the Institute for Drug Control (Shanghai, China, #111590) and dissolved in dimethyl sulfoxide (DMSO). Doxorubicin was purchased from Sangon Biotech (Shanghai, China, #DB3456) and dissolved in ultra-purified water. FITC Annexin V and propidium iodide were obtained from BD Biosciences (Franklin Lakes, USA, #556420). The cell cycle detection kit was from Key GENBioTECH (Nanjing, China). Antibodies against PARP (#9542), BAX (D2E11, #5023), BCL-2 (#2876), Vimentin (5G3F10, #3390), C-Myc (D84C12, #5605), GSK-3β (27C10, #9315), phosphor-GSK-3β (#8213), active β-catenin (D13A1, #8814) and β-catenin siRNA II (#6238) were from Cell Signaling Technology (Danvers, USA). Trypsin-EDTA, Lipofectamine 2000 (#11668) and TRIzol reagent (#15596) were from Invitrogen (Rockville, MD, USA). CHIR99021 (#SML1046) and antibody against β-actin (#A2228) were from Sigma-Aldrich (Saint Louis, USA). Monoclonal antibody of rabbit anti-Ki-67 and RIPA Cell Lysis Buffer (#P0013B) were got from Beyotime Institute of Biotechnology (Shanghai, China). SuperSignal Chemiluminescent HRP Substrate (#34078) was from Thermo Fisher scientific Inc (Rockford, USA). Matrigel (#356234) was purchased from BD Biosciences (Bedford, USA). PrimeScript RT reagent kit (#RR037A) and SYBR Premix Ex Taq II (#RR420L) were bought from TaKaRa (Dalian, China).

### Cells and culture

The human osteosarcoma cells of 143-B and HOS were obtained from American Type Culture Collection (ATCC, Manassas, VA, USA). 143-B cells were grown in modified Eagle medium (MEM) and HOS cells were grown in Dulbecco minimum essential medium (DMEM), supplemented with 10% fetal bovine serum (FBS), penicillin (100 units/ml), and streptomycin (100 μg/ml), at 37 °C in a humidified atmosphere of 5% CO_2_.

Patient osteosarcoma primary cells were prepared from the human osteosarcoma specimen. Briefly, the freshly harvested surgical osteosarcoma resection from clinical patient was soaked in sterile PBS with penicillin (500 units/ml) and streptomycin (500 μg/ml) for 10 min, and washed 3 times with sterile PBS without penicillin and streptomycin. The osteosarcoma tissue was then cut into small pieces (about 1 mm^3^) and placed on the tissue culture dish for 5 min before DMEM medium (containing 10% FBS, 100 units/ml penicillin and 100 μg/ml streptomycin) was supplied. The tissues were cultured for 3–10 days until the osteosarcoma primary cells sticked to the bottom of the tissue culture dish. The patient osteosarcoma primary cells were then cultured as 143-B and HOS cells.

### Cell transfection

Briefly, 143-B cells were co-transfected with either small interfering RNA-targeting β-catenin (100 nM si-β-catenin) or 100 nM si-control for 72 h, using Lipofectamine 2000 (Invitrogen). β-catenin siRNA II (#6238) was bought from Cell Signaling Technology (Danvers, USA). si-control was designed and produced by Shanghai GenePharma Co.,Ltd (Shanghai, China).

### Cell viability assay

Cell viabilities of the 143-B and HOS cells, patient osteosarcoma primary cells were assessed in real time using an xCELLigence RTCA DP system (xCELLigence-Real-Time Cell Analyzer, Roche Applied Science, Mannheim, Germany).

Briefly, during the xCELLigence RTCA DP system analysis, the baseline was determined using 100 µl of medium before loading 3000 cells into each well of an xCELLigence–E-plate (Roche Applied Science, Mannheim, Germany). Cells were exposed to PPI at the concentrations of 0.2–1.6 µM, the largest concentration (1/10,000 V/V) of DMSO included in the solvent of PPI was added in the wells of the control group and used as negative control. And 0.5 μM doxorubicin (DOX) was used as positive control. Cell proliferation was monitored continuously for 48 h by recording the cell index (CI) for each well at all time points. CI is a measure of the surface area covered by the cells and can be used to quantify the number of cells attached to the sensors in the E-plate.

All the experiments were performed in triplicate and the means of the results were used for optimization.

### Cell apoptosis analysis

143-B or HOS cells were seeded into 12-well plates at a density of 1 × 10^5^ cells/ml for 24 h. Then PPI was added to the cells at the final concentration of 0.4, 0.8, 1.6 μM, and DMSO (1/10,000 V/V) only was added in the control group and used as vehicle control.

Cells were double-stained with Annexin V-FITC and PI Apoptosis Detection Kit, after being challenged with PPI for 24 h, according to the manufacturer’s description. Briefly, harvested cells were washed twice with pre-cooled PBS and incubated with 5 μl of FITC-labeled Annexin-V in the dark room for 30 min at room temperature, followed by the addition 5 μl of propidium iodide (50 μg/ml) in the dark room for 5 min on ice. Early apoptotic (Annexin V-FITC stained only) and late apoptotic (Annexin V-FITC and propidium iodide double stained) cells were detected by flow cytometry (Accuri C6, BD Biosciences, USA) and combined for analysis.

### Cell cycle analysis

143-B or HOS cells were cultured in 12-well plates at a density of 1 × 10^5^ cells/ml for 24 h. PPI was added into the cells at the final concentration of 0.4, 0.8, 1.6 μM, DMSO (1/10,000 V/V) only was added in the control group and used as vehicle control, then the cells were incubated for 24 h. The cell cycle distribution was analyzed using the Cell Cycle Detection kit according to the manufacturer’s instructions. In brief, incubated cells were fixed with cold 70% ethanol overnight and washed with cold PBS. The fixed cells were incubated for 30 min with 40 μl of RNaseA at 37 °C, followed by another 30 min of incubation with 160 μl of propidium iodide in dark at 4 °C. Stained cells were applied to a flow cytometer (Accuri C6, BD Biosciences, USA). The cell cycle distribution was analyzed using the Modifit Software and showed as the percentage of cells containing G_0_/G_1_, S, and G_2_/M DNA content, as determined by propidium iodide staining.

### Western blot analysis

The total proteins were extracted from 143-B or HOS cells challenged with 0.8 μM of PPI for different times. Collected cells were washed twice with pre-chilled PBS, then re-suspended in RIPA Cell Lysis Buffer and kept on ice for 30 min. The supernatant of cell lysate was collected after centrifuging at 13,000 g and 4 °C for 15 min. The protein concentration was measured by the bicin choninic acid (BCA) method. To determine the levels of target protein expression, equal amounts of proteins were separated by SDS-PAGE and transferred to PVDF membranes, blotted with primary antibodies respectively, and detected using the SuperSignal Chemiluminescent HRP Substrate after incubation with peroxidase-conjugated secondary antibodies for 1 h at room temperature. Images of the Western blots were acquired using the ChemiDoc MP imaging System (Bio-Rad, USA) and the software Image Lab version (Bio-Rad, USA).

### Migration and invasion assay

Migration and invasion of 143-B and HOS cells were determined using an xCELLigence RTCA DP system. In each well of the designed CIM-Plate 16, a total volume of 165 μl medium containing 10% FBS was loaded into the lower chamber and 40 μl medium without FBS was loaded to the upper chamber. The pore size was 8 μM. An additional 100 μl of cell suspension with 40,000 143-B cells or 30,000 HOS cells and indicated concentration of PPI were loaded into each of the upper chambers. The total volume of medium in each upper chamber was 140 μl, and the final concentration of PPI in the upper chambers was 0 (DMSO, 1/10,000 V/V), 0.4, 0.8, or 1.6 μM, respectively. Cell migration was then continuously monitored every 15 min for a period of 24 h.

Cell invasion assay for 143-B and HOS cells was performed using the same conditions as used in the migration assay, except the upper chambers were pre-coated with matrigel (1:40 dilution) and the cell invasion was continuously monitored for a period of 48 h.

Migration of the 143-B cells was also determined by the wound healing assay. Briefly, logarithmic phase 143-B cells were seeded at a density of 1 × 10^5^ cells/ml in 6-well plates. A straight line was marked using a marker pen on the back of the well with pretreated cells. Another straight line, perpendicular to the one on the back of each well at the center, was gently scraped using the tip of disposable pipette, when the 143-B cells were grown to 90% confluence. The wells were then rinsed with sterile PBS for 3 times and filled with serum-free MEM medium. The wound healing was observed and photographed at the time point of 0 and 24 h with 0.8 μM of PPI. The migrated distance was calculated by subtracting the average distance between wound edges from that at the beginning. Experiment was performed at least triplicate.

### Quantitative analysis of mRNA levels

Quantitative real-time PCR was used to detect β-catenin expression in matched cancerous tissues and adjacent noncancerous tissues from 3 patients. Total RNA was extracted using the TRIzol reagent according to the manufacturer’s protocol. The levels of β-catenin were examined using the forward primer, 5-AGACGGAGGAAGGTCTGAGG-3, and the reverse primer, 5-GGCCATGTCCAACTCCATCA-3. Reverse transcription PCR was done using the PrimeScript RT reagent kit. RT-PCR was performed using SYBR Premix Ex Taq II and the Bio-Rad CFX 96 (Richmond, CA, USA) real-time PCR system. GAPDH levels were used as internal controls, and fold changes were calculated using the 2^−ΔΔCt^ method. Each experiment was performed in triplicate.

### Immunohistochemistry

Xenograft tumors were resected immediately and fixed in 10% neutral buffered paraformaldehyde for 24 h. Selected samples were embedded in paraffin and sectioned. Sections (5 μm thick) were deparaffinized, rehydrated with PBS (pH 7.4), incubated with aqueous 3% H_2_O_2_ for 10 minutes, and then the antigen was retrieved in 0.1% trypsin (M/V) for 10 minutes at 37 °C. Samples were blocked with 5% BSA at RT for 30 min, then monoclonal antibody of rabbit anti-Ki-67 was applied at the concentration of 1:150, at 4 °C overnight. Next, they were incubated with second antibody conjugated Diaminobenzidine (DAB) at RT for 15 minutes and then washed with PBS. DAB was applied for 5 minutes and then slides were counterstained with hematoxylin. Sections were then mounted with neutral gum after transparency with xylene. Imagine were obtained by imagine autoanalysis system (Olympus BX50) and one representative area was shown.

### *In vivo* model establishment and treatment

Four-week-old male BALB/c nude mice, weighing 18–21 g, were purchased from Shanghai SLAC Laboratory Animal Co, Ltd. (Shanghai, China). Mice were maintained in separate cages at the Animal Experiment Centre of Shanghai University of Traditional Chinese Medicine. Mice were kept in a pathogen-free environment, maintained under a 12 h-light/dark cycle, as well as with free access to water and food. All procedures and animal experiments used in this study were approved by the Animal Care and Use Committee, Shanghai University of Traditional Chinese Medicine. All animal experiments, including *in vivo* model establishment and treatment, xenograft tumor resection for tumor weight and tumor volume analysis, and immunohistochemistry assay, in this study were performed following the regulation (SZY2016004) on using animals for laboratory experiments by the Animal Care and Use Committee, Shanghai University of Traditional Chinese Medicine.

Animal experiment was performed to evaluate orthotopic tumor growth of osteosarcoma cells *in vivo*. In brief, logarithmic phase 143-B cells were harvested by trypsin-EDTA digestion, washed twice with PBS, and re-suspended in matrigel at a final concentration of 2 × 10^7^ cells/ml. A total of 10 μl cell suspension was injected into one of the proximal tibia of each anesthetized nude mouse. A total of 30 mice inoculated osteosarcoma cells were then randomly divided into 3 groups, PPI group, DOX group and control group (n = 10). After a 24 h inoculation, mice in PPI group were intraperitoneally injected with 100 μl PPI (4 mg/kg·body weight), mice in DOX group were intraperitoneally injected with 100 μl DOX (1 mg/kg·body weight), while mice in control group were injected with same volume of saline including DMSO (1/10000 V/V) for 28 continuous days, once a day. The tumor formation and survival of mice was observed every day. Tumor volumes were measured every 7 days and body weights were measured every 3 days. The growth of tumor in the bone was monitored by X-ray imaging using the *In-Vivo* Multispectral System (Carestream Health Canada Company, Ontario, Canada) every week. Mice were sacrificed after continuous administration for 28 days, and tumors were isolated and weighed. The long diameter (a) and short diameter (b) of individual orthotopic tumor of each mouse were measured with calipers. The volume (V) of the orthotopic tumor was calculated according to the formula: V = 1/2 × a × b^2^. Survival rate of mice in each group was calculated using the equation: survival rate of mice = number of mice alive/original number of mice.

### Ethics statement

The protocol of this study was approved by the human research ethics committee of Longhua Hospital, Shanghai University of Traditional Chinese Medicine (2013LC52). The study was performed in accordance with the principles of the Declaration of Helsinki. All the 3 participants provided written informed consent, whose cancerous tissues and adjacent noncancerous tissues were used for β-catenin mRNA expression detection by quantitative real-time PCR, according to the regulation on using cancerous tissues and adjacent noncancerous tissues for laboratory experiments by the human research ethics committee of Longhua Hospital, Shanghai University of Traditional Chinese Medicine.

### Statistical analysis

All data are representative of at least three independent experiments. The results shown in this study are presented as mean ± standard deviation (SD). Differences between the experimental groups were assessed by one-way ANOVA using SPSS 18.0 software. The statistical significance of differences was indicated as ^*^ *p* < 0.05, ^**^ *p* < 0.01 and ^***^ *p* < 0.001.

## Electronic supplementary material

Supplementary Information
