# Supplementary material for: Polyphyllin I suppresses human osteosarcoma growth by inactivation of Wnt/β-catenin pathway in vitro and in vivo
Source: Sci Rep. 2017 Aug 8;7:7605. doi: 10.1038/s41598-017-07194-9 (PMC5548759; doi:10.1038/s41598-017-07194-9)
Supplement: Supplementary file 1 — Supplementary Information [file 41598_2017_7194_MOESM1_ESM.doc]

Polyphyllin I suppresses human osteosarcoma growth by inactivation of Wnt/β-catenin pathway *in vitro* and *in vivo*

Junli Chang^1,2,3#^, Yimian Li^1,2,3#^, Xianyang Wang^1,2,3#^, Shaopu Hu^1,2,3^, Hongshen Wang^1,2,3^, Qi Shi^1,2,3^, Yongjun Wang^1,2,3,4*^ and Yanping Yang^1,2,3*^

Authors’ Affiliations:

^1^Longhua Hospital, Shanghai University of Traditional Chinese Medicine, Shanghai, 200032, China; ^2^Spine Institute, Shanghai University of Traditional Chinese Medicine, Shanghai, 200032, China; ^3^ Key laboratory of theory and therapy of muscles and bones, Ministry of Education; ^4^School of Rehabilitation Science, Shanghai University of Traditional Chinese Medicine, Shanghai, 201203, China.


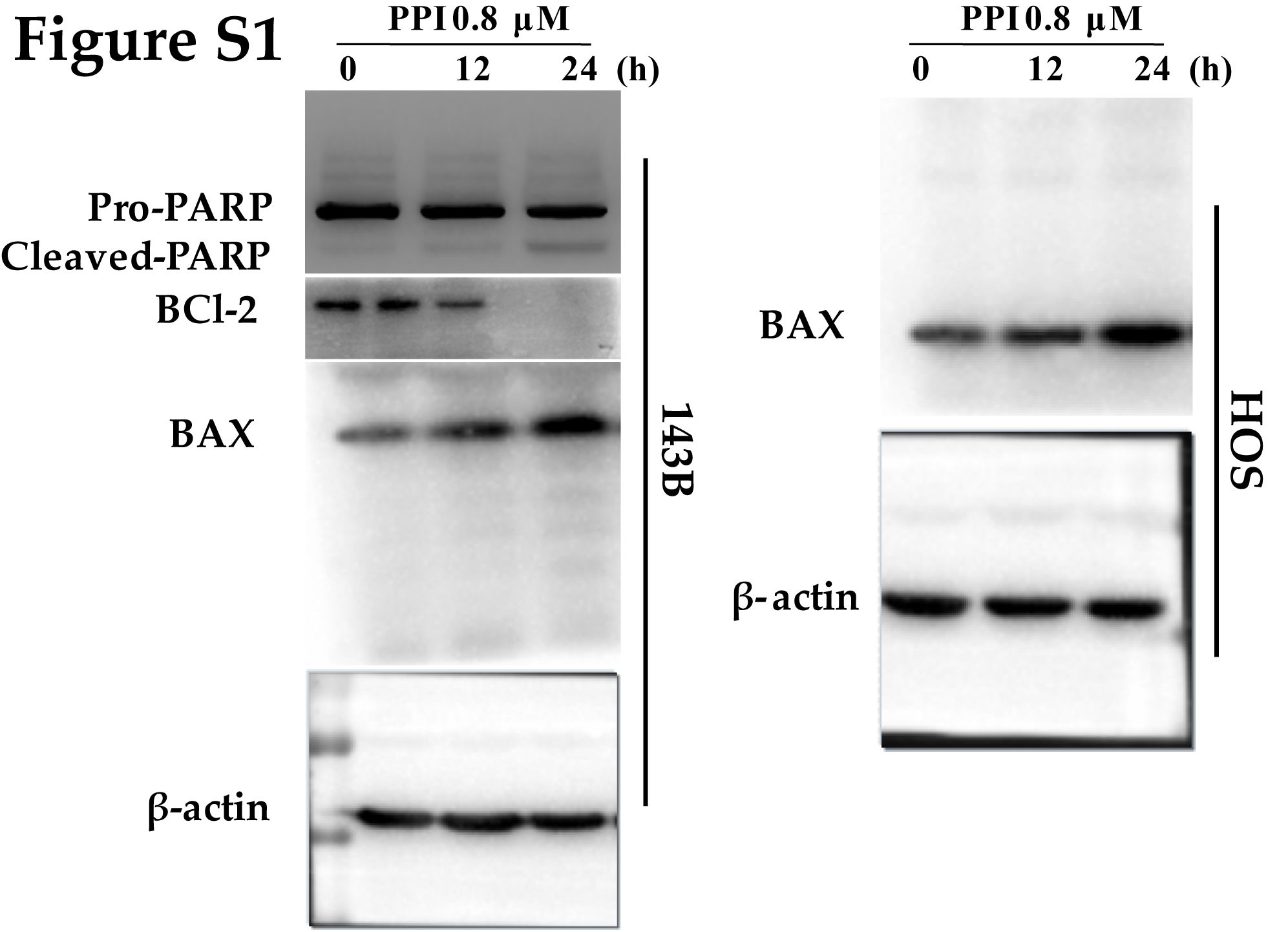


Figure S1. The full-length blots of Figure 2E.


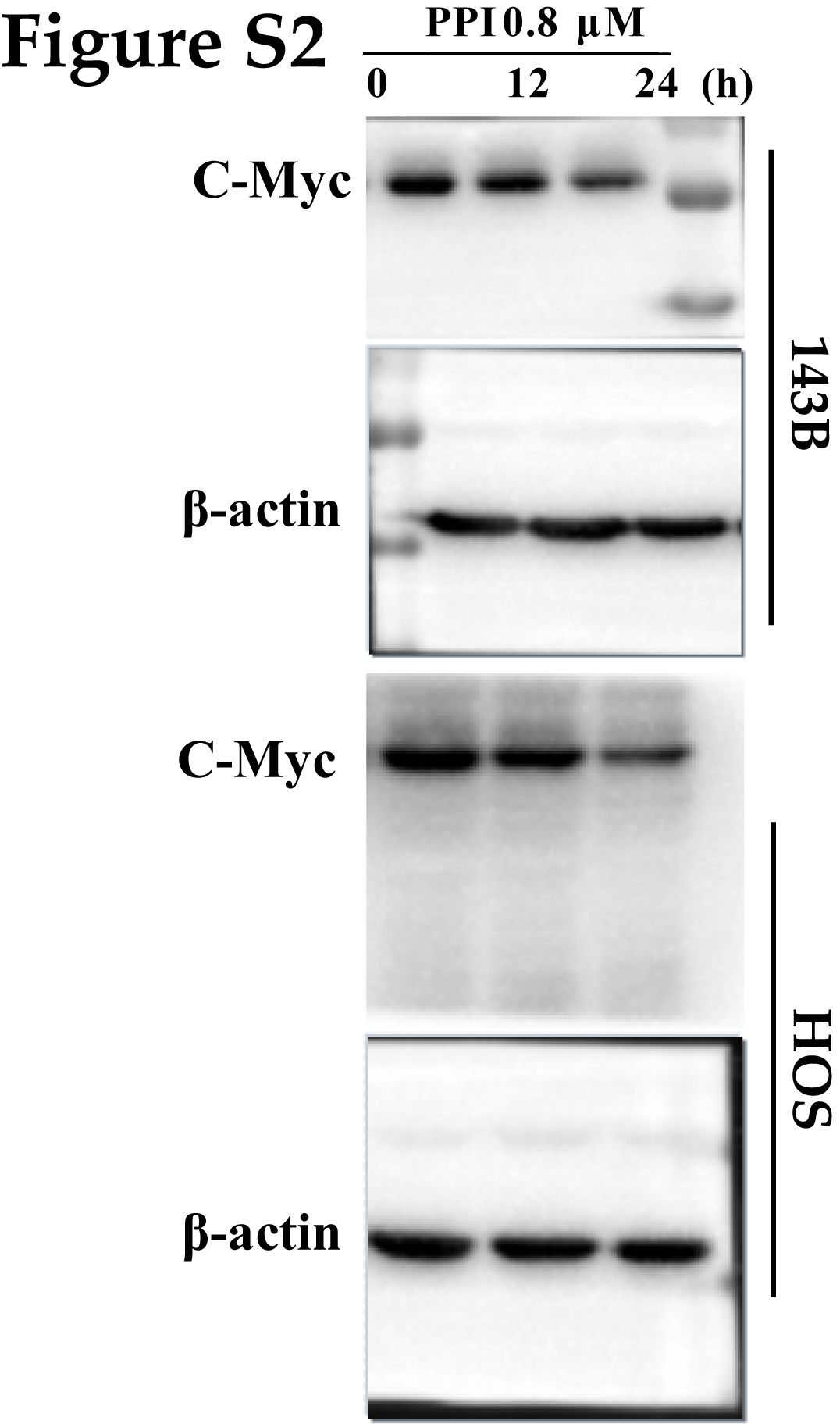


Figure S2. The full-length blots of Figure 3E.


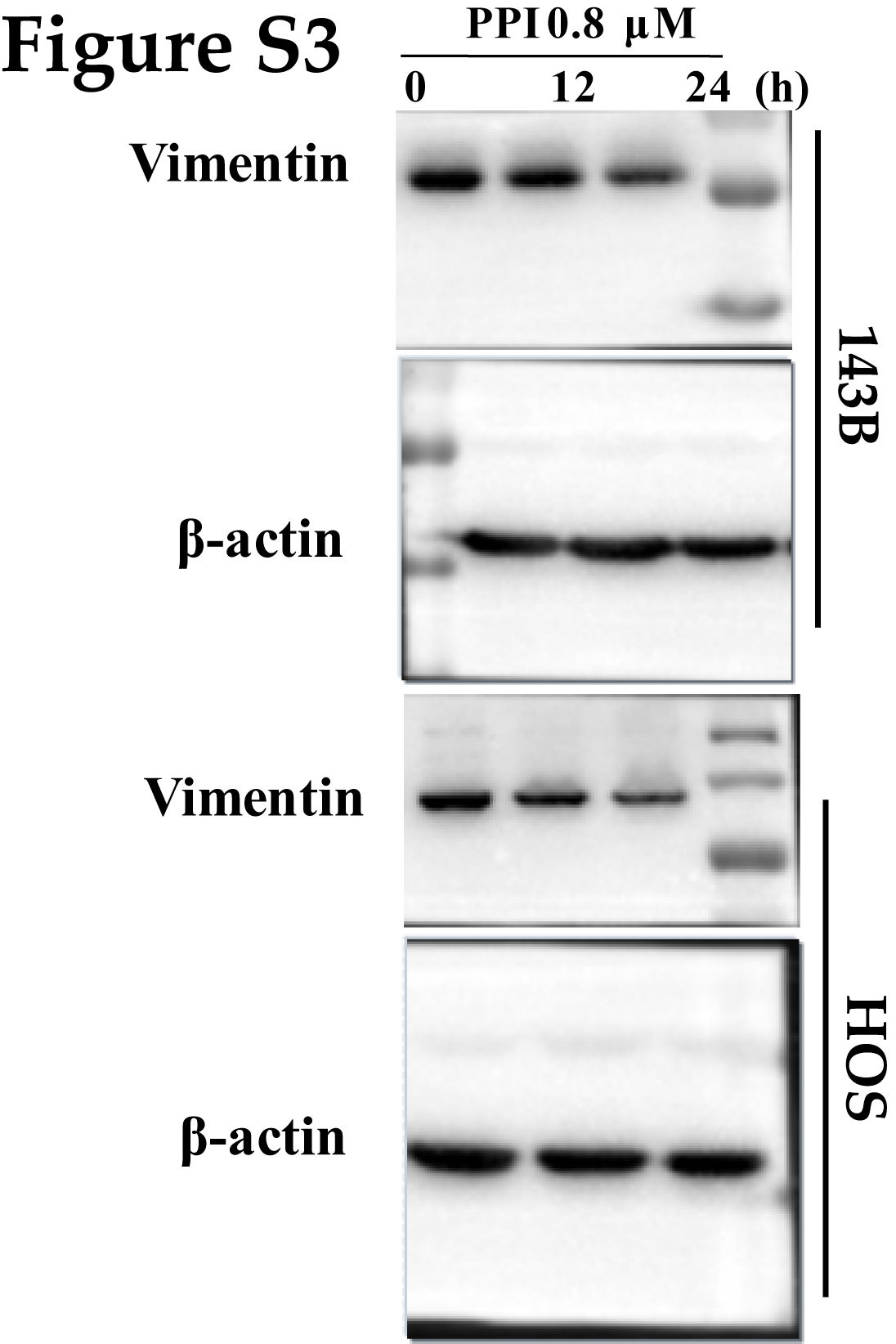


Figure S3. The full-length blots of Figure 4G.


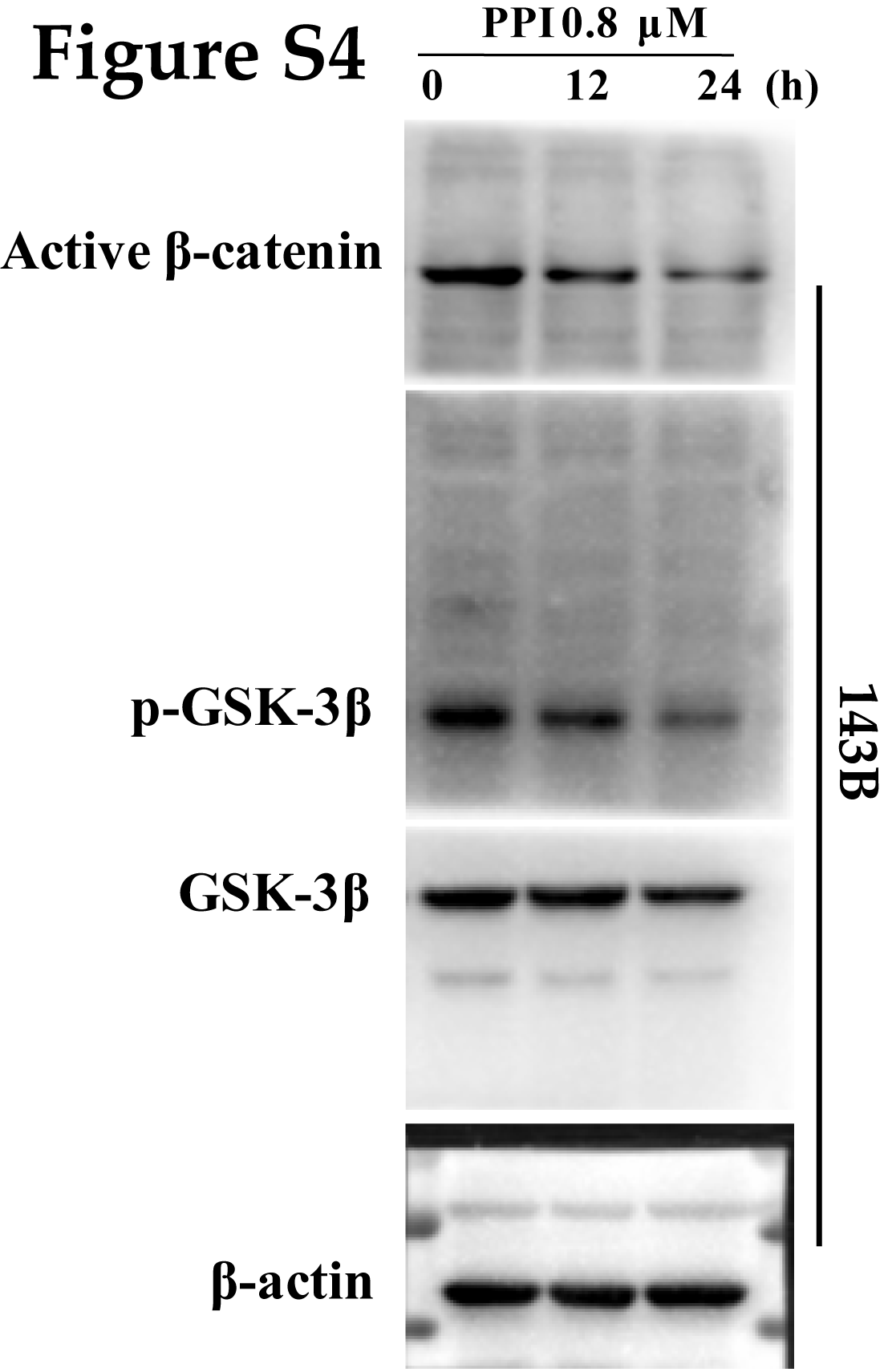


Figure S4. The full-length blots of Figure 5B.


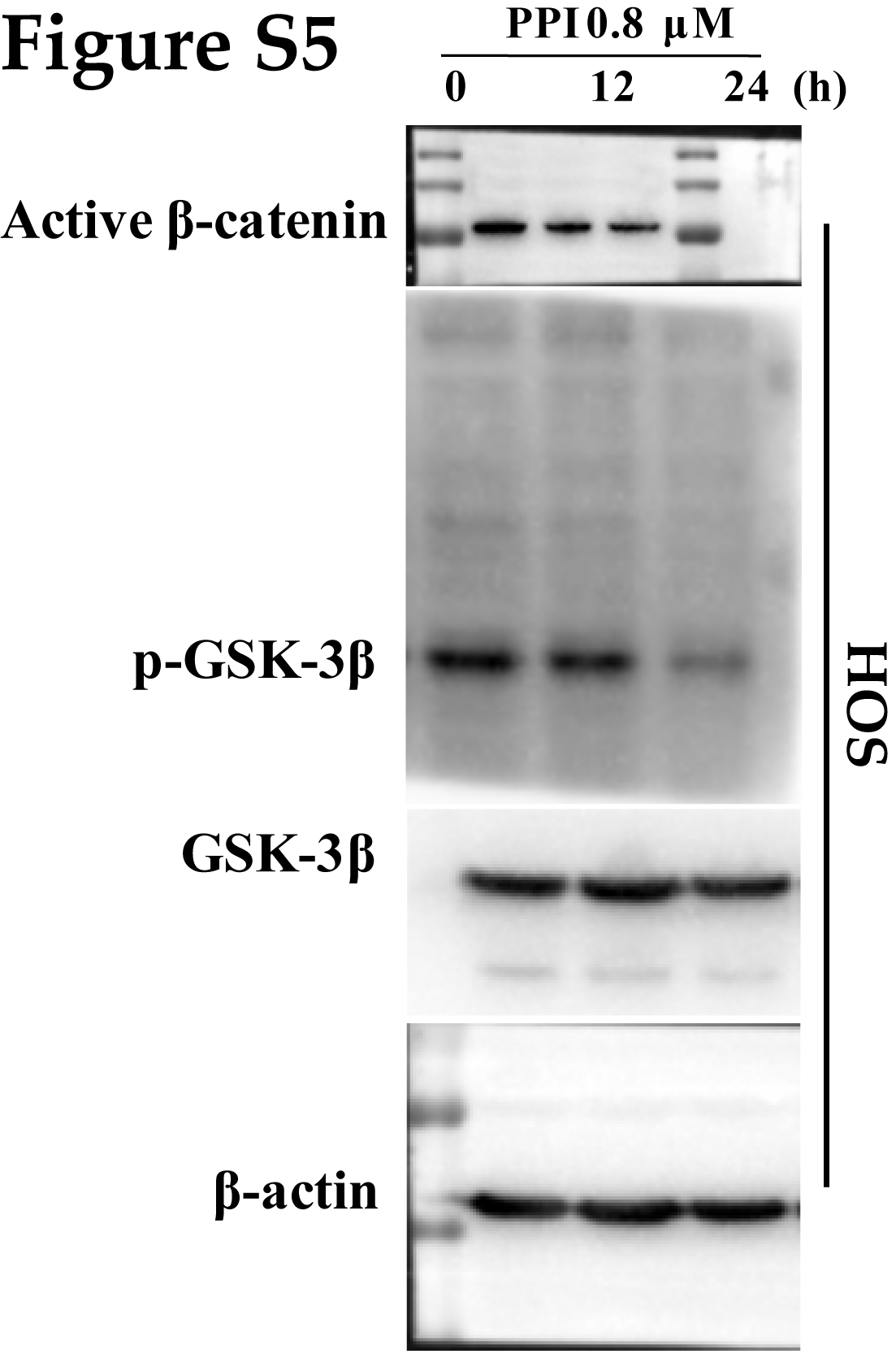


Figure S5. The full-length blots of Figure 5C.


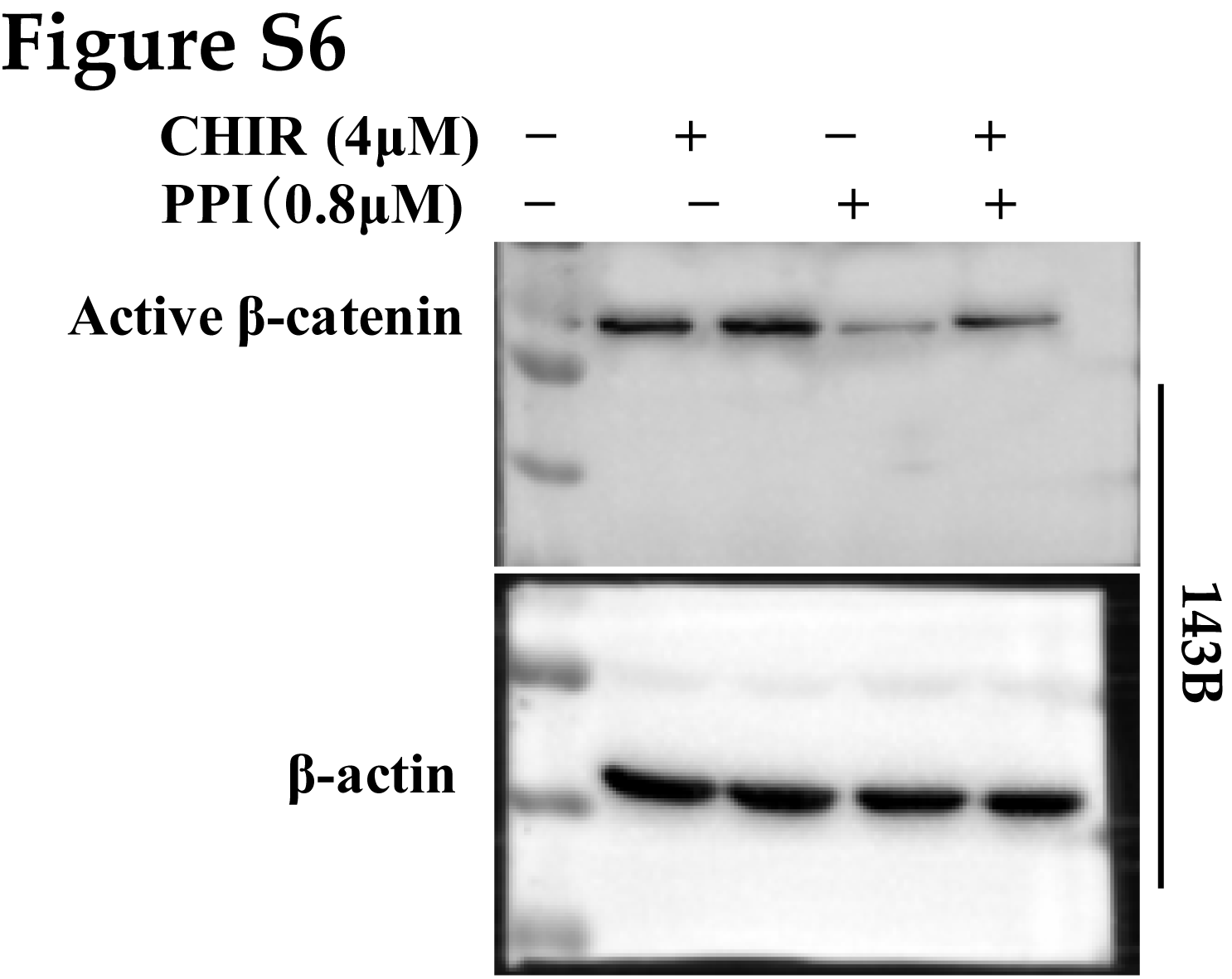


Figure S6. The full-length blots of Figure 5D.


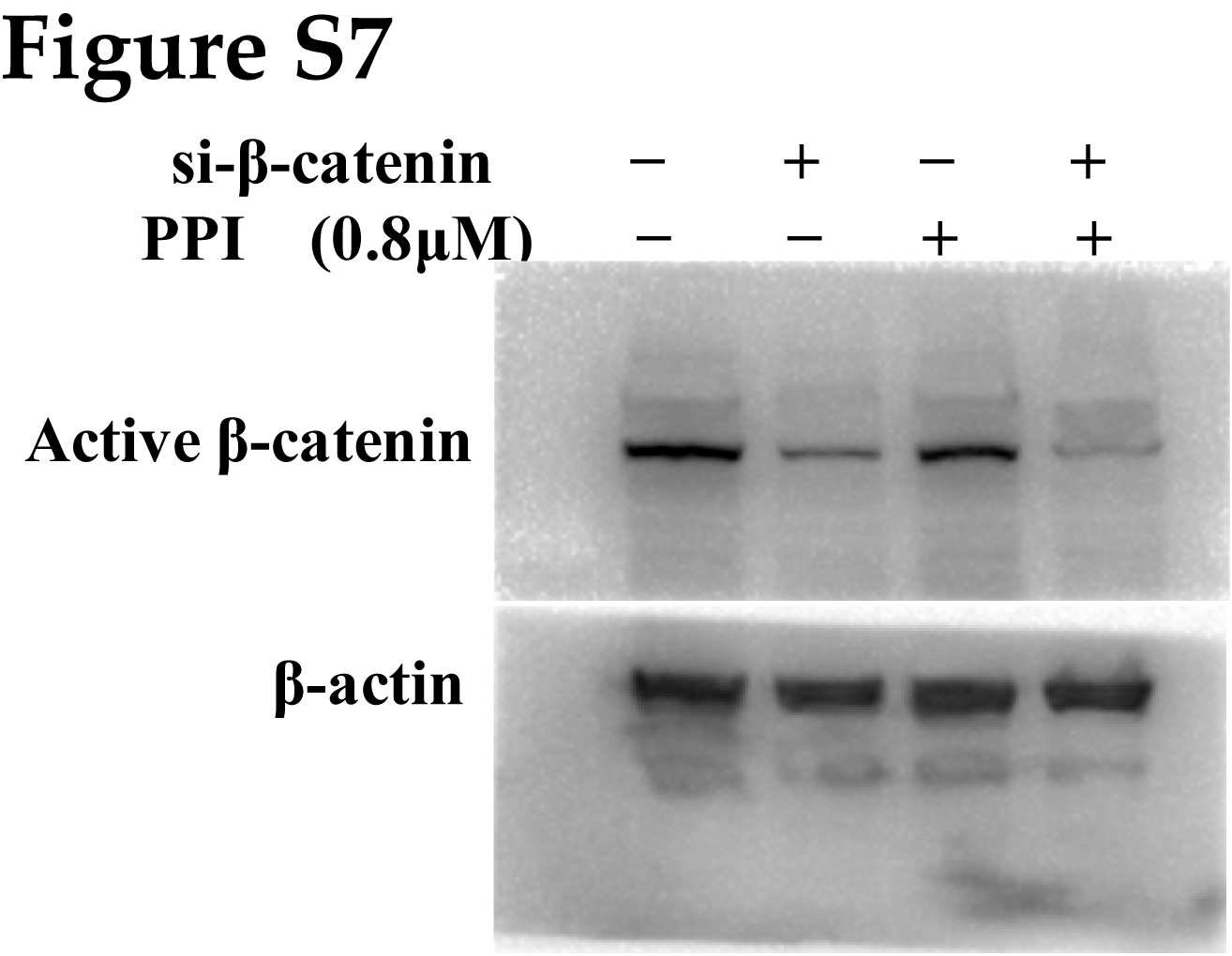


Figure S7. The full-length blots of Figure 5F.
